# Optimization of ultrasound‐assisted solvent extraction of hemp (*Cannabis sativa* L.) seed oil using RSM: Evaluation of oxidative stability and physicochemical properties of oil

**DOI:** 10.1002/fsn3.1796

**Published:** 2020-07-27

**Authors:** Reza Esmaeilzadeh Kenari, Bahareh Dehghan

**Affiliations:** ^1^ Department of Food Science and Technology Faculty of Agricultural Engineering Sari Agricultural Sciences and Natural Resources University Sari Iran

**Keywords:** Behnken design, box, hemp seed oil, optimization, response surface methodology, ultrasonic‐assisted extraction

## Abstract

In this study, the effect of solvent ratio of hexane to isopropanol (0:100, 50:50, and 100:0 v/v%), extraction temperature (30, 45, and 60°C), and time (30, 60, and 90 min) were investigated on the oil extraction efficiency, total phenol content, DPPH radical scavenging, peroxide value, and oil color index. Extraction conditions were optimized by response surface methodology and Box–Behnken design. The optimal conditions were obtained as hexane‐to‐isopropanol ratio of approximately 3:2, temperature of 40.26°C, and ultrasonication time of 54.40 min. Then, the hemp seed oil was extracted under the optimal conditions. The optimal predicted contents for oil yield (31.22%), total phenolics (3.19 mg GA/g oil), DPPH inhibition (73.86%), peroxide (4.62 meq/kg), and color index (28.2) were agreed with the predicted conditions because the RSE values were less than 5%. Hemp seed oil can be used extensively due to its high nutritional values, and antioxidant potential and ultrasound can improve oil extraction as a simple and fast method.

## Graphical abstract

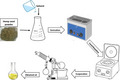

## 1 INTRODUCTION

The hemp (*Cannabis sativa* L.) plant, as a rich resource of the bioactive compounds, has been used for pharmaceutical purposes and preparing healthy food (Palade et al., 2019). Hemp is an annual plant containing more than 480 compounds that divided into different photochemical classes in which cannabinoids are the most studied (Drinić et al., 2018). On average, hemp seed contains 25%–35% oil, 20%–25% protein, 20%–30% carbohydrate, and 10%–15% insoluble fiber (Wang, Tang, Yang, & Gao, 2008). Two unsaturated essential fatty acids, linoleic acid (omega 6) and α‐linolenic acid (omega 3), approximately constitute 50%–70% and 15%–25% of the total fatty acid content in the plant, respectively (Haji‐Moradkhani, Rezaei, & Moghimi, 2019). The hemp seed oil is considered as the only plant oil with the optimal ratio (3:1) of omega‐6 to omega‐3, which plays an important role in promoting the human health through reducing diseases such as sclerosis, high blood pressure, high cholesterol levels, and cancer (Da Porto, Natolino, & Decorti, 2015).

In addition, hemp seeds have a lot of natural antioxidants such as phenolic compounds, phytosterols, and tocopherols, which may play a role in reducing the risk of chronic diseases, including cancer, lipid metabolism, dermatological diseases, and cardiovascular health, and prevent the oxidation of unsaturated fatty acids (Irakli et al., 2019).

Proper pretreatment of seeds before the oil extraction is one of the most important steps for producing a high‐quality oil with a high efficiency (Bakhshabadi, Mirzaei, Ghodsvali, Jafari, & Ziaiifar, 2018). Therefore, the extraction technique with new methods of pretreatment has been receiving more attention. To achieve an easy and efficient extraction is the purpose of these methods. An efficient extraction process is characterized by lower amounts of solvents required, shorter extraction times, and lower temperatures used, as well as increased efficiency and overall cost‐effectiveness (Li, Pordesimo, & Weiss, 2004).

In food processing, the advantages of using ultrasound include faster transferring of mass and energy, decreased temperature, extraction selectivity, reduced size of equipment, faster responses to control of the extraction process, and increased production (Chemat & Khan, 2011). Based on the development and design of extraction processes, ultrasound extraction is also considered as a green technique because it uses lower levels of energy and provides high‐quality and safe products by using alternative natural solvents (Rombaut, Tixier, Bily, & Chemat, 2014). The diffusion of ultrasound waves in a solvent containing oilseed causes easy extraction as a result of the cavitation phenomenon. When the cavities collapse at the cell surfaces, the cell walls are broken by the strike; therefore, the main components outflow rapidly into the solvent (Li et al., 2004); (Jalili, Jafari, Emam‐Djomeh, Malekjani, & Farzaneh, 2018). Ultrasound waves can generate a series of sequential contractions and expansions in the environment (sponge effect). Forces generated by this mechanical mechanism can be higher than surface tension, which holds the solvent within the capillary tubes of the tissue, creating microscopic channels in the oilseeds that removes oil. This process makes oil extraction easily; thus, the extraction process can be conducted at low temperatures (Fernandes, Linhares, & Rodrigues, 2008).

Jalili et al. (2018) showed that the oxidative stability of the extracted canola seed oil was increased with increasing ultrasound process. Also, oil extraction by ultrasound method had a higher yield compared with the Soxhlet method.

Moghimi, Farzaneh, and Bakhshabadi (2018) studied the ultrasound‐assisted process for black cumin oil extraction. The results showed that oil extraction efficiency was increased with increasing time and power of ultrasound. All oil samples had an identical and unchanged refractive index, but the acid value, peroxide value, color index, and total phenol content of oil were increased by ultrasound treatment.

The purpose of this study was to examine the parameters including time, temperature, and different solvents at a constant frequency of 35 kHz by using the ultrasound device and their effects on oxidative stability of the hemp seed oil to increase the efficiency of oil extraction.

## 2 MATERIALS AND METHODS

### 2.1 Preparation of samples

Industrial hemp seed contains no or very little tetrahydrocannabinol (von Bayern, Heathcote, Rutz, & Kacelnik) (Khorasan variety, containing 34.1% oil content and 7.5% moisture content as determined by Soxhlet and oven drying, respectively) was prepared from Oilseeds Development Company. Preparation process included phases of cleaning, sifting, and drying of seeds in an oven at the temperature of 60°C for 6 hr until achieving the moisture contents of 3% (Hernández‐Santos et al., 2016). Then, the samples were milled and passed through a sieve with 300‐μm mesh.

### 2.2 Ultrasound‐assisted oil extraction

In a glass Erlenmeyer flask, 50 g of milled hemp seeds was separately mixed with 150 ml of various solvents including isopropanol, hexane, and a ratio 50:50 of hexane/isopropanol (Azadmard‐Damirchi, Habibi‐Nodeh, Hesari, Nemati, & Achachlouei, 2010). The mixture of hemp seed/solvent were treated by ultrasonic bath (Elma S‐30‐H, Germany) at the frequency of 35 kHz and power of 800 W for 30, 60, and 90 min at 30, 45, and 60°C.

At the end of the extraction process, the fluid extracted was separated from the solid residues by filter cloth and remaining solids were washed twice with the relevant solvent, then centrifuged by the centrifuge (HS1028, Iran) for 5 min, 1,792 *g* at 25°C. The collected solution was concentrated in vacuum rotary evaporator (IKA RV‐10, Germany) at 37°C. The obtained oil was poured after weighing into laboratory glasses and stored in a freezer at −18°C for the next experiments (Jalili et al., 2018).

### 2.3 Efficiency of oil extraction

In order to determine the efficiency of oil extraction, weights of the used seeds and the obtained oil were determined. Then, the efficiency of oil extraction was calculated by using the following equation (Samaram et al., 2015) (Eqn 1).

(1) Efficiencyofoilextraction(%)=(Weightofextractedoil/Primaryweightofseeds)×100

### 2.4 Measurement of the peroxide value (PV)

In a flask, 5 g hemp oil was mixed with 30 ml mixture of 3:2 v/v acetic acid: chloroform. Then, 0.5 ml saturated potassium iodide (KI) was added to the solution and kept in the dark for one minute. Next, 30 ml of distilled water was added and the sample titration was carried out with sodium thiosulfate (0.01 N) until the solution became colorless (Maghsoudlou, Esmaeilzadeh Kenari, & Raftani Amiri, 2017). The peroxide value (mEq of oxygen/kg) was calculated using the following equation:

(2) $$\text{PV}=\frac{(V_{2}‐V_{1})\times N\times 1,000}{m}$$

where V_2_ and V_1_ are the sample and control titration numbers, respectively, N is the sodium thiosulfate normality, and m is the sample weight in gram.

### 2.5 Total phenolic compounds

First, 2.5 g hemp oil was dissolved in 2.5 ml hexane and vortexed for 1 min. 2.5 ml solvent of methanol: water (80:20) was added to the solution and centrifuged at 2,800 *g* for 5 min, and then, the subnatant solution was transferred to a 50‐ml balloon (repeated three times). 2.5 ml Folin–Ciocalteu solution and then 5 ml sodium carbonate 7.5% were added to the solution, and the final volume of 50 ml was obtained by adding distilled water. The samples were stored overnight. Finally, the absorbance values of the samples were read using a spectrophotometer at 765 nm (Capannesi, Palchetti, Mascini, & Parenti, 2000).

### 2.6 DPPH radical scavenging activity (%)

The mixture of 0.1 ml hemp oil with 2.9 ml fresh methanolic solution of DPPH (60 μM) was shaken and placed in dark condition at room temperature for 30 min. The absorbance of the samples was read by the spectrophotometer at 517 nm. The percentage of radical scavenging DPPH was calculated by the following equation (Li, Xu, et al., 2016):

(3) $$\text{DPPH}\;\%=\left[\left({\text{Blank}}_{\text{abs}}‐{\text{Sample}}_{\text{abs}}\right)/{\text{Blank}}_{\text{abs}}\right]\;\;\times \;\;100$$

### 2.7 Determining the oil color index

The spectrophotometry method was used to evaluate the oil coloration as a mixture of colors. To this purpose, light absorbance of the oil was measured at wavelengths of 460, 550, 620, and 670 nm. Then, the color index was measured through the following equation in lovibond yellow color (Moghimi et al., 2018):

(4) $$\text{Color}\;\text{index}=1.29\;A_{460}+69.7\;A_{550}+41.2\;A_{620}‐56.4\;A_{670}$$

### 2.8 Statistical analysis

The response surface methodology (RSM) is employed to find the optimal mode of interaction between the factors and estimate the optimal conditions for the extraction process with the minimum number of experiments required. The software RSM determines the effects of independent variables on processes, individually or in a combined manner. In this study, the effects of independent variables including the ratio of solvent (A), temperature (B), and time (C) on the response variables including the efficiency of extraction (Y_1_), total phenol content (Y_2_), percentage of DPPH radical scavenging (Y_3_), peroxide value (Y_4_), and color index of oil (Y_5_) were examined. Table 1 represents the coded and actual values of the independent variables used in this process. The Box–Behnken design including 15 experiments with 3 replications (to calculate the process reproducibility) was applied for the statistical analysis. The applied treatments and obtained response variables are presented in Table 1. Finally, the response variables were fitted and the best conditions were selected. Statistical analysis was performed by Design‐Expert software version 7.

**Table 1:** Box–Behnken design applied from RSM for hemp seed oil extraction

Run	Independent variables			Responses				
	Solvent ratio (v/v%)	Temperature (°C)	Time (min)	Oil yield (%)	Total phenol (mg GAE/g)	DPPH inhibition (%)	Peroxide (meq/kg)	Color index
1	0	−1	−1	23.8	2.84	65	1.8	30.4
2	−1	−1	0	26	3.49	76.8	3	43.5
3	0	1	−1	22.8	2.5	60.2	4.5	34.3
4	0	0	0	31.7	3.24	76.1	6	35.3
5	0	0	0	31.5	3.39	75.8	6	35
6	1	1	0	26.2	2	53.5	6.9	8.3
7	−1	0	−1	23.3	3.04	71.5	2.7	41.2
8	0	0	0	31.6	3.4	76.5	5.4	32.3
9	1	0	1	30	2.2	54.5	9	−3.1
10	1	0	−1	26.4	2.05	54.9	2.4	−0.8
11	0	−1	1	28	2.74	67.2	7.5	31.9
12	0	1	1	25	2.6	59	9.6	36.8
13	1	−1	0	27.2	2.15	58.7	3.9	4
14	−1	1	0	23.2	2.95	69.7	6.3	49.4
15	−1	0	1	27.2	3.3	75.2	7.6	46.6

Independent variables	Unit	Symbol	Levels		
			−1	0	+1
Solvent ratio (hexane: isopropanol)	v/v %	A	0:100	50:50	100:0
Temperature	°C	B	30	45	60
Time	min	C	30	60	90

## 3 RESULTS AND DISCUSSION

Table 2 shows the results of the derived analysis of variance from examining the factors influencing responses. The table includes sums of squares and the probability of significance for linear effects (A: solvent ratio, B: temperature, and C: time), quadratic expressions (A^2^, B^2^, C^2^), and the interaction effects of the variables (AB, AC, BC). According to the table, factors with a probability value of less than 5% have a significant effect; the more the value is close to 0, the higher the effect of the factor will be. The values of lack of fit were not significant for none of the tests performed (*p* > .05). This implied the selected models were suitable to demonstrate the data trends. The final model presented for the intended responses is shown in Table 3.

**Table 2:** Analysis of variance for the studied independent variables in oil extraction by ultrasound treatment

Source	Yield extraction (%)		Total phenol (mg GAE/g)		DPPH scavenging activity (%)		Peroxide value (meq/kg)		Color index	
	Sum of squares	*p* > *F*	Sum of squares	*p* > *F*	Sum of squares	*p* > *F*	Sum of squares	*p* > *F*	Sum of squares	*p* > *F*
Model	134.97	<.0001*	3.78	0.0003*	1,100.10	<.0001*	78.41	<.0001*	4,215.09	<.0001*
A–solvent ratio	12.75	.0001*	2.40	<0.0001*	640.82	<.0001*	0.84	.1124	3,710.91	<.0001*
B–temperature	7.61	.0004*	0.17	0.0082*	80.01	<.0001*	15.40	<.0001*	45.12	.0224*
C–time	24.15	<.0001*	0.021	0.1975	2.31	.0656	62.16	<.0001*	6.30	.2773
AB	0.81	.0406*	0.038	0.1021	0.90	.2021			0.64	.7137
AC	0.023	.6665	3.025E−003	0.5973	4.20	.0249*			14.82	.1205
BC	1	.0284*	1.000E−002	0.3524	2.89	.0467*			0.25	.8178
A^2^	15.71	<.0001*	0.48	0.0009*	97.61	<.0001*			379.45	.0002*
B^2^	55.80	<.0001*	0.42	0.0012*	147.32	<.0001*			18.49	.0912
C^2^	29.21	<.0001*	0.42	0.0012*	179.20	<.0001*			35.20	.0346*
Residual	0.54		0.048		2.09		3.12		21.21	
Lack of fit	0.52	.0553	0.032	0.4609	1.85	.1714	2.88	.3023	15.75	.3601
Pure error	0.02		0.016		0.25		0.24		5.46	
Core total	135.51		3.83		1,102.19		81.53		4,236.30	

* The values are significant at *p* < .05.

**Table 3:** Designed equation models for the selected dependent variables

Responses	Equation	*R* ^2^	*R* ^2^‐adj	C.V
Oil yield	Y = 31.60 + 1.26A – 0.98B + 1.74C + 0.45AB – 0.075AC – 0.5BC – 2.06A^2^ – 3.89B^2^ – 2.81 C^2^	0.9960	0.989	1.22
Phenol	Y = 3.34 – 0.55A – 0.15B + 0.051C + 0.098AB ‐ 0.027AC + 0.05BC – 0.36A^2^ – 0.34B^2^ – 0.34C^2^	0.9723	0.9223	5.34
DPPH	Y = 76.13 – 8.95A – 3.16B + 0.54C + 0.48AB – 1.03AC – 0.85BC – 5.14A^2^ – 6.32B^2^ – 6.97C^2^	0.9957	0.9880	0.9415
Peroxide	Y = 5.51 + 0.32A + 1.39B + 2.79C	0.9617	0.9513	9.67
Color index	Y = 34.20 – 21.54A + 2.37B + 0.89C – 0.4AB – 1.92AC + 0.25BC – 10.14A^2^ + 2.24B^2^ – 3.09C^2^	0.9950	0.9860	7.27

### 3.1 Influence of operational parameters on the efficiency of oil extraction

According to Table 1, the efficiency of oil extraction was varied in a range from 22.8% to 31.7%. The highest efficiency was achieved in a condition including the time of 60 min, and the temperature of 45°C with a mixture of 50:50 ratio hexane/isopropanol solvent. All linear effects, quadratic expressions, and interactions between factors (except AC) had significant effects (*p* < .05) on oil extraction rate (Table 2).

**Figure 1:**
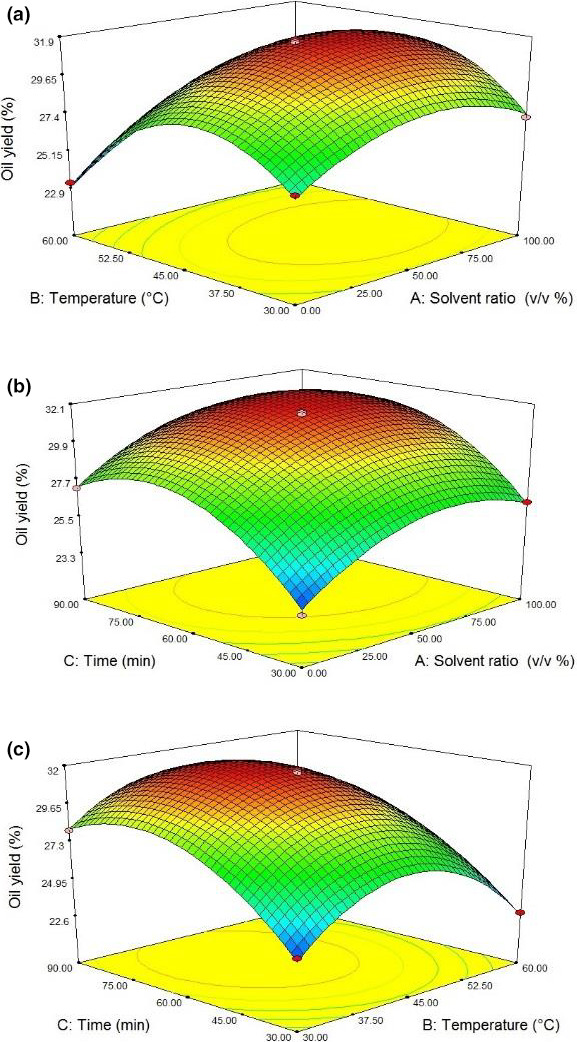
Response surface plots of oil yield (%) at interaction factors of (a) solvent ratio and temperature, (b) solvent ratio and time, and (c) temperature and time

According to Figure 1, the type of solvent also influenced the rate of oil extraction from hemp seeds. Oil extraction by hexane solvent was more efficient than isopropanol individually. Li, Qu, Zhang, and Wang (2012) reported that using a combined solvent led to the higher affinity between lipophilic compounds and the solvent than employing hexane and isopropanol individually. In another study, it was observed that adding polar solvents (isopropanol) to nonpolar solvents (hexane) increased the recovery percentage of polar lipids such as phospholipids and lipoproteins and thus the total fat content (Hernández‐Santos et al., 2016). Since various lipids show differences in polarity, one solvent would be insufficient to extract them. Within the cell membrane, polar lipids (phospholipids and glycolipids) are often attached to protein molecules through strong bonds of hydrogen or electrostatic types. Thus, adding a polar solvent such as isopropanol leads to denaturation of the proteins, as well as breaking the bonds between lipids and proteins before the solvent extraction by hexane. Therefore, adding alcohol to nonpolar solvents highly increases the final amount of lipid extracted. Although the hexane easily dissolves neutral lipids, micelle‐like structures prevent the extraction of high lipid contents. The solution is often adding alcohol to help them degrade (Halim, Gladman, Danquah, & Webley, 2011).

As shown in Figure 1, increasing the temperature to 45°C led to increased efficiency of oil extraction. A further increase in temperature beyond 45°C causes decrease in response.

This result was similar to reports by Senrayan and Venkatachalam (2019) that they explained the reason for the increase in oil extraction with increasing temperature was due to the higher solubility of the oil in the solvent. In fact, the increased temperature was enhanced solvent penetration into the seeds due to decreased solvent viscosity and increased kinetic energy; therefore, the extraction process was accelerated. Decreased oil extraction at higher than a critical temperature is accounted for by that the steam pressure is increased and more bubbles are created, but due to lower pressure difference between the inside and outside of the bubbles, they collapse with less intensity (Gutte, Sahoo, & Ranveer, 2015).

Based on Figure 1, it could be concluded that increasing the ultrasound time up to 60 min enhanced the efficiency of oil extraction. Longer duration of ultrasound treatment increases the cavitation time. Consequently, more cell walls are destroyed, enhanced the contact surface between the oilseeds and the solvent. This leads to more oil released from the solid structure and increased the extraction rate (Luque‐Garcıa & De Castro, 2004). As observed, a further increase in extraction time resulted in decreased efficiency of oil extraction because a considerable amount of solvent is lost due to evaporation over a longer period, and the remaining solvent would not be sufficient for efficient extraction. Another possible cause of oil degradation is prolonged exposure to heat during a long time (Palconite et al., 2018).

### 3.2 Influence of operational parameters on total phenol content

The range of total phenol content of oil was 2–3.49 (mg GAE/g) (Table 1), which is consistent with the results of Smeriglio et al. (2016). According to Table 2, some linear coefficients (A and B) and all quadratic coefficients (A^2^, B^2^, and C^2^) were significant in the model developed for phenol (*p* < .05). However, the linear effect of time (C) and the interactions of variables (AB, AC, and BC) were not significant (*p* > .05). Moreover, factors with high coefficients featured higher influences on the variable studied (Palconite et al., 2018). As seen from Table 3, the solvent type parameter with a higher coefficient (0.55) had more effect on phenol content compared with the other parameters. Solvent polarity can influence the phenolic compounds extracted (Tab araki & Nateghi, 2011). Different solvents can make differences in the profile of phenolic compounds, leading to the different antioxidant activity of the intended compound (Li et al., 2012). In the present study, the hemp oil extracted by isopropanol solvent contained higher phenol compounds compared with the oil extracted by the hexane solvent. Based on the theory "like dissolves like,” in fact, more proportion of the polar phenolic compounds may be extracted (Li, Xu, et al., 2016). Isopropanol is a polar solvent, unlike hexane, leading to more efficient extraction of polar phenolic compounds than it (Uoonlue & Muangrat, 2019).

**Figure 2:**
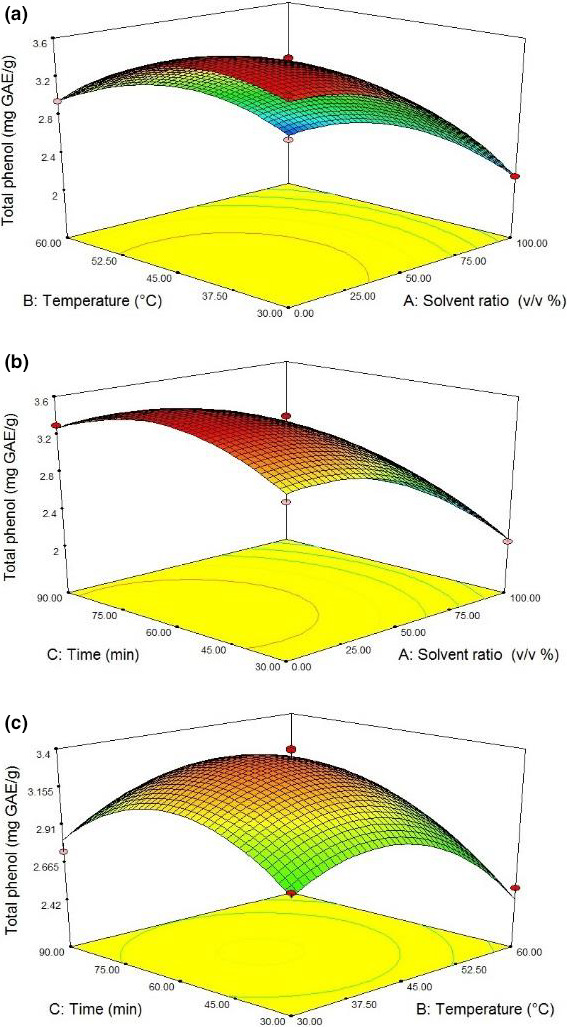
Response surface plots of total phenol (mg GAE/g) at interaction factors of (a) solvent ratio and temperature, (b) solvent ratio and time, and (c) temperature and time

According to Figure 2, increasing temperature up to 45°C enhanced the phenolic content extracted due to increased mass transfer. In fact, mild heating could soften plant tissues and reduce the cell wall resistance, leading to hydrolysis of bonds between phenolic compounds and cellular components (phenol–protein or phenol–polysaccharide). As a result, the solubility of phenolic compounds would increase and higher phenolic content would be extracted. Movement of the active compounds from the substrate might occur to a certain level of temperature. This was possibly due to the degradation of these compounds at higher temperatures; when would be applied longer times, the antioxidant activity might be decreased due to this degradation. In this regard, Senrayan and Venkatachalam (2019) reported similar results, pointing out that bioactive compounds are heat‐sensitive. The similar results were also reported by Yilmaz and Toledo (2004), and Irshaid and Mansi (2009) reported a decreased extraction rate of phenolic compounds observed at high temperatures was due to the polymerization reactions between the compounds.

As shown in Table 3, the time component positively influenced on phenol content. Figure 2 shows that increasing the time of the ultrasound process up to 60 min enhanced the total phenol content of the oils to 3.49 (mg GAE/g). As a result of increased time, the contact surface between the solvent and the solid material would expand, further destroying cell walls and thus allowing the higher mass transfer. This meant higher levels of releasing the phenolic compounds into the solvent. As shown in Figure 2, at a constant temperature of 45°C, the phenol content decreased from 3.4 to 2.2 (mg GAE/g) with increasing time from 60 to 90 min. This was because longer times led to solvent evaporation and reduced extraction rate, which is similar to the results of Senrayan and Venkatachalam (2019).

### 3.3 Influence of operational parameters on DPPH radical scavenging

According to Table 2, all the terms (except for the linear effect of time and the interaction of solvent × temperature) significantly influenced DPPH radical scavenging (*p* < .05). The solvent type variable, having the highest coefficient (8.95), showed the highest effect on DPPH inhibition. The results indicated that using the polar solvent isopropanol, compared with the nonpolar solvent (hexane), led to increased DPPH inhibition. Consistent with our results, Chen et al. (2016) demonstrated that the antioxidant activity of oil was increased with increasing the solvent polarity. In other words, the extracted oil would contain higher antioxidant compounds. The DPPH radical scavenging activity was correspondent to the phenol content. The extracted oil using isopropanol, compared with the extracted oil using hexane, showed higher levels of DPPH radical scavenging activity due to the higher phenol content. The similar result was reported by Uoonlue and Muangrat (2019). The high antioxidant activity in the extracted oil with mixture solvent of hexane and isopropanol resulted from the small compounds with antioxidant activity occurring in the oil (Jalili et al., 2018).

**Figure 3:**
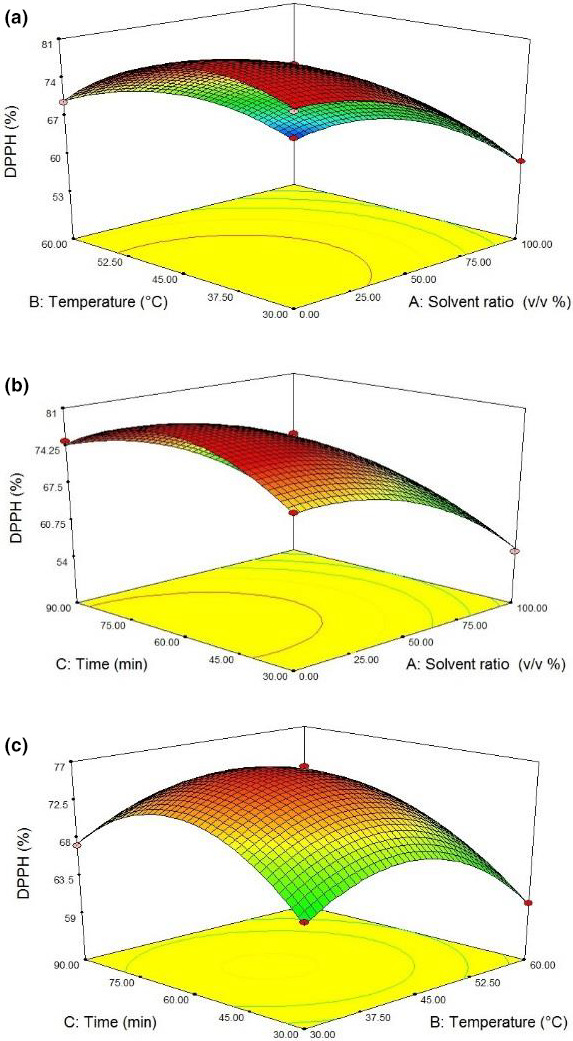
Response surface plots of DPPH scavenging activity (%) at interaction factors of (a) solvent ratio and temperature, (b) solvent ratio and time, and (c) temperature and time

According to Figure 3, increasing the temperature up to 45°C firstly led to increased antioxidant activity. Using a warm environment could facilitate the recovery of fat‐soluble antioxidants, resulting in enhanced antioxidant activity. Increased temperature enhanced solvent extraction both through increasing the diffusion coefficient and through enhancing the solubility of phenolic compounds (Al‐Farsi & Lee, 2008). However, the antioxidant activity decreased with increasing the temperature from 45 to 60°C. Kishk and El Sheshetawy (2010) reported similar outcomes that the free DPPH radical scavenging activity was increased with a temperature increment from 20 to 54°C. This was followed by a decreasing trend because of degradation of antioxidant compounds. The decreased oxidative stability was probably due to decreased contents of tocopherol and antioxidant compounds due to increased heat.

The time variable had a positive effect on DPPH scavenging activity, when applied individually (Table 3). It might be attributed to the fact that longer times made possible time for antioxidant compounds to dissolve in the extraction solvent. Moreover, plant cells exposed to ultrasound waves might be undergone the timer required to be completely degraded, releasing the compounds into the solvent.

### 3.4 Influence of operating parameters on the peroxide value

The peroxide range of oil was as 1.8–9.6 (meq/kg) (Table 1). The peroxide content of the oil samples was less than 10, indicating the oil extracted with good quality and no bad taste. Based on the analysis of variance (Table 2), of the studied parameters, temperature (B) and time (C) had a significant effect on oil peroxide content (*p* < .05), but the solvent type showed no significant effect on peroxide (*p* > .05). The peroxide value was slightly increased by hexane, but it was lower when using isopropanol individually or in combination with hexane. This could be explained based on the phenol content in the oil. More phenolic compounds were extracted by isopropanol, leading to higher oxidative stability of the oil. Consistent with the present results, Jalili et al. (2018) reported that the using of a mixture of isopropanol and hexane solvents and the reactions between phenolic compounds and hydroxyl radicals during cavitation led to the production of hydroxylated polyphenolic compounds with higher antioxidant activities than with the natural phenolic compounds.

**Figure 4:**
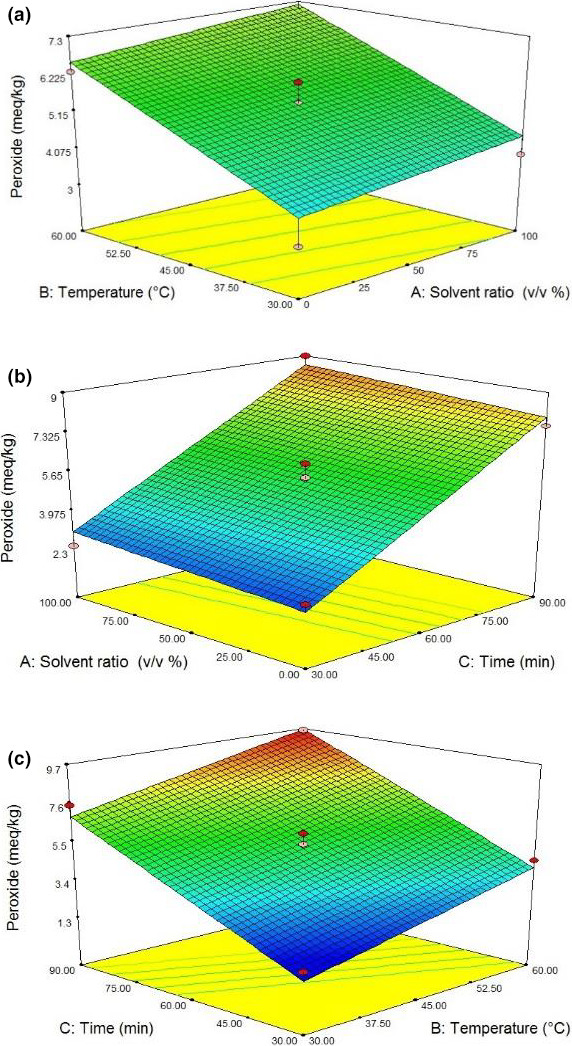
Response surface plots of peroxide (meq/kg) at interaction factors of (a) solvent ratio and temperature, (b) solvent ratio and time, and (c) temperature and time

As shown in Figure 4, at a specific time (30 min) and in a certain type of solvent (a mixture of hexane and isopropanol), the oil peroxide value was increased from 1.8 to 4.5 (meq/kg) with increasing the temperature from 30 to 60°C. Lipolytic enzymes, located just under the thin shells of the seeds, are not able to attack the fats inside intact cells. However, these enzymes launch their activity because high temperatures cause physical changes in the cells. Certainly, the increased acidity results from the heating‐induced breakdown of the ester bonds in triglyceride molecules. Jalili et al. (2018) reported that increasing the temperature had a direct significant effect on degradation of phenolic compounds and producing the free radicals, thereby reducing the oxidative stability of the extracted oil.

The ultrasonication time, with a high coefficient (2.79), had the highest effect on oil peroxide value. In other words, increasing the time increased the rate of oil oxidation due to the production of free radicals once the ultrasound‐induced cavities were collapsed, consequently increasing the peroxide value of oil samples. The similar result was reported by Jalili et al. (2018).

### 3.5 Influence of operational parameters on the color index

**Figure 5:**
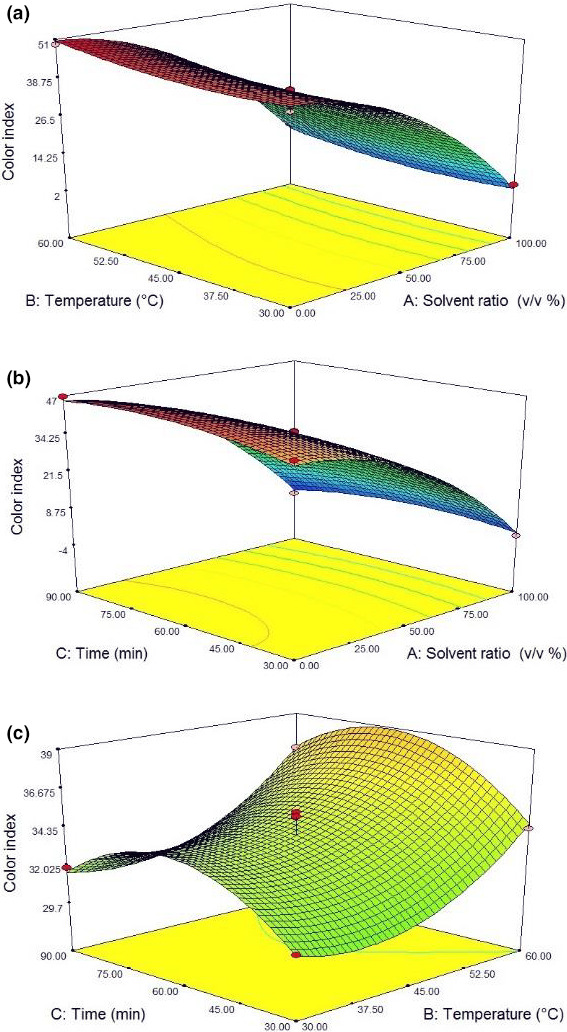
Response surface plots of color index at interaction factors of (a) solvent ratio and temperature, (b) solvent ratio and time, and (c) temperature and time

**Figure 6:**
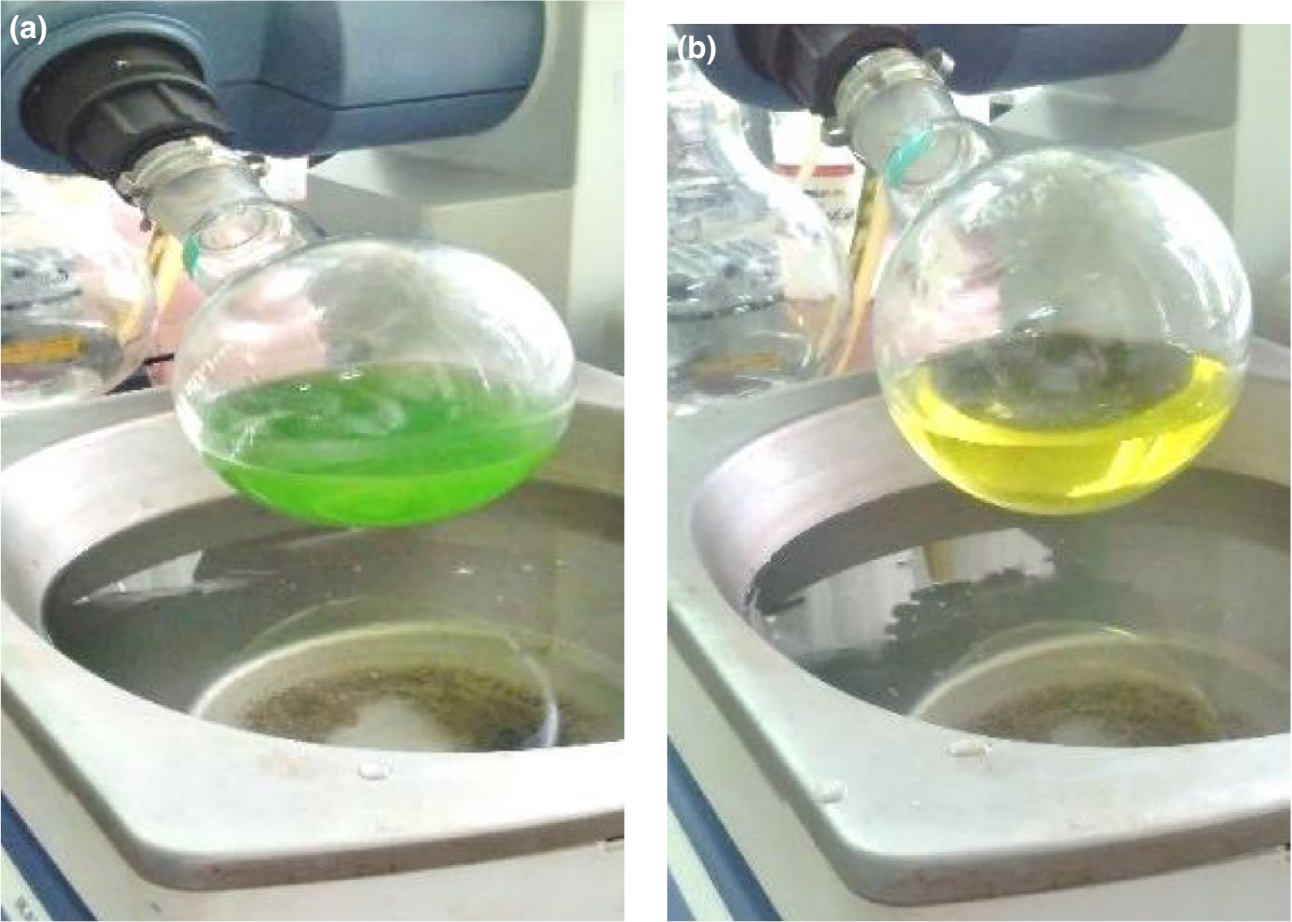
Images of different color of hemp seed oil extracted with (a) isopropanol and (b) hexane solvents (before evaporation of solvent)

**Figure 7:**
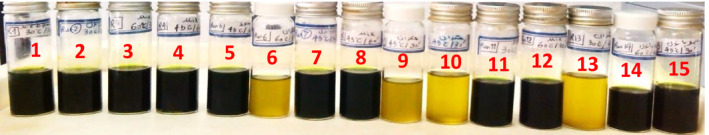
Appearance of extracted hemp seed oil under different conditions, after evaporation of solvent (arranging of treatments according to Table 1)

The dark green coloration of the hemp oil is due to containing high amounts of the important pigments of chlorophyll a and b. Carotenoids are also contained in the hemp oil. According to Table 2, only the linear effect of solvent ratio (A), temperature (B), and the quadratic effect of the solvent type (A^2^) and time (C^2^) on oil color index were significant (*p* < .05). The solvent type variable showed the highest effect on the color index. As shown in Figure 5, the oil color index decreased with a steep slope when the extraction solvent was changed from isopropanol to hexane. As shown in Figure 6, the oil extracted by the hexane solvent is yellow. However, using the isopropanol solvent individually or in combination with hexane turned the oil color to dark green as a result of the high extracted chlorophyll contents into the oil. Finally, after rotary evaporation of the solvent, concentrated oil had higher color intensity due to the increased chlorophyll concentration (Figure 7). Although the chlorophyll pigments are not water‐soluble, they are easily dissolved in organic solvents such as ethanol, acetone, ether, and chloroform. Many previous studies have shown that the efficiency of 95% could be achieved in chlorophyll extraction by using organic solvents (such as ethanol and acetone as polar ones). Organic solvents are capable of dissolving the fat‐soluble compounds (Li, Xu, et al., 2016 ).

Previous studies have indicated that cell break downs caused through ultrasound shock significantly enhanced the efficiency of chlorophyll extraction with organic solvents (Hosikian, Lim, Halim, & Danquah, 2010). Increasing the temperature and time of ultrasound increased the oil color index. This was attributed to the further break downs in seeds and further releasing of pigments such as chlorophyll into the oil. Li et al. (2004) reported that the formation of brown substances in heat‐treated compounds is the result of a variety of non‐enzymatic browning reactions of Millard type, caramelization, and degradation of phospholipids.

### 3.6 Confirmation of models

As Table 4 information suggests, low differences between the values predicted by the software as optimal conditions and the actual values obtained in the laboratory conditions implied the accuracy of experiments performed and optimization conditions. In fact, RSE values less than ± 5 were considered in accordance with the predicted values, proving suitability of the models (Sulaiman et al., 2017).

(5) $$\text{Residual}\;\text{standard}\;\text{error}\;\left(\text{RSE}\right)\;\left(\%\right)=\frac{\left(\text{Actual}\;\text{value}‐\text{Predicted}\;\text{value}\right)}{\text{Predicted}\;\text{value}}\;\;\times \;\;100$$

**Table 4:** Predicted and actual response values in the optimum extraction conditions

	Solvent ratio (hexane: propanol) (v/v %)	Temperature (°C)	Time (min)	Oil yield (%)	Total Phenol (mg GAE/g)	DPPH inhibition (%)	Peroxide (meq/kg)	Color index
Predicted	61.18:38.82 ~ 3:2	40.26	54.40	31.22	3.19	73.86	4.62	28.2
Actual	3:2	40	55	32.10	3.21	76.55	4.54	29.01
RSE (%)				2.81	0.62	3.64	1.73	2.87

Values of the coefficient of determination (*R* ^2^) indicate the percentage of changes determined by the model made for each response. The closer the value is to 1, the better the model will perform in predicting the responses. A high *R* ^2^ value indicates the high accuracy of the model. In the present study, *R* ^2^ value was above 0.96 for of all responses, confirming the models examined. Also, the coefficient of variation (CV) is a measure of the data distribution; low values of CV indicate the reliability of experiments and practical works conducted. The CV value of our responses was <10 (Table 3).

### 3.7 Optimal condition of extraction variables

In order to achieve the maximum levels of oil extraction efficiency and antioxidant activity, and the minimum peroxide value and color index, optimization of oil extraction from hemp seed with ultrasound pretreatment was conducted at different temperatures (30–60°C) and different times (30–90 min) using different solvents (hexane, isopropanol, and a mixture of hexane/isopropanol). The results indicated that the purposes mentioned were achieved with the ultrasound time of 54.40 min, the temperature of 40.26°C, and hexane‐to‐isopropanol ratio of approximately 3–2 (v/v%) (Table 4). Therefore, the desirability of 0.702 was achieved. The actual values were well consistent with the predicted values because the RSE values for the optimal conditions were <5%.

## 4 CONCLUSION

The results indicated that RSM was completely effective and reliable to determine the optimal conditions for extracting the hemp seed oil. Moreover, increasing the temperature and time of the ultrasound process led to increased efficiency of oil extraction, phenolic contents, and DPPH radical scavenging, followed by a decrement in them. However, the peroxide value tended to linearly increase at all times. With increasing temperature, the color index was first decreased and then increased. The response was in contrast to the time. The solvent type had significant effects on all responses except the peroxide value. Using the mixed solvents resulted in higher levels of efficiency of oil extraction, total phenol content, and DPPH radical scavenging compared with applying hexane and isopropanol as individual solvents. However, the oil had higher color index when extracted by isopropanol (polar solvent) than hexane (nonpolar solvent) or the combined solvent.

## CONFLICT OF INTEREST

The authors have declared no conflict of interest.

## ETHICAL APPROVAL

This study does not involve any human or animal testing.
